# Improvement Critical Care Patient Safety: Using Nursing Staff Development Strategies, At Saudi Arabia

**DOI:** 10.5539/gjhs.v7n2p335

**Published:** 2015-01-12

**Authors:** Enas M. Bassuni, Magda M. Bayoumi

**Affiliations:** 1 Nursing Administration Department, College of Applied Medical Sciences, King Khalid University, KSA; 2 Nursing College, Cairo University, Egypt; 3 Medical Surgical Department, Nursing College-Al Farabi Colleges for Dentistry and Nursing, KSA

**Keywords:** patient, safety, critical care, nursing staff

## Abstract

Intensive care units (ICUs) provide lifesaving care for the critically ill patients and are associated with significant risks. Moreover complexity of care within ICUs requires that the health care professionals exhibit a trans-disciplinary level of competency to improve patient safety. This study aimed at using staff development strategies through implementing patient safety educational program that may minimize the medical errors and improve patient outcome in hospital. The study was carried out using a quasi experimental design. The settings included the intensive care units at General Mohail Hospital and National Mohail Hospital, King Khalid University, Saudi Arabia. The study was conducted from March to June 2012. A convenience sample of all prevalent nurses at three shifts in the aforementioned settings during the study period was recruited. The program was implemented on 50 staff nurses in different ICUs. Their age ranged between 25-40 years. Statistically significant relation was revealed between safety climate and job satisfaction among nurses in the study sample (p=0.001). The years of experiences in ICU ranged between one year 11 (16.4) to 10 years 20 (29.8), most of them (68%) were working in variable shift, while 32% were day shift only. Improvements were observed in safety climate, teamwork climate, and nurse turnover rates on ICUs after implementing a safety program. On the heels of this improvement; nurses’ total knowledge, skills and attitude were enhanced regarding patient safety dimensions. Continuous educational program for ICUs nursing staff through organized in-service training is needed to increase their knowledge and skills about the importance of improving patient safety measure. Emphasizing on effective collaborative system also will improve patient safety measures in ICUS.

## 1. Introduction

Intensive care settings provide lifesaving care for the critically ill patients and are associated with significant risks for adverse events and serious errors with multiple interactions occurring between health multidisciplinary health care providers, patients, and medical devices with increasingly complex interface (Chang, Multz, & Hall, 2005; Welters et al., 2011). However the medical errors can occur during any of these interactions. Especially with critically ill patients are highly vulnerable to medical errors, because they usually have both co- morbidities and acute organ dysfunctions (Rainey & Combs, 2011). As well as, the life-sustaining treatments and highly technical routine care used in intensive care units (ICUs) expand many opportunities for medical errors, in addition complexity of care within ICUs requires that the health care professionals exhibit a trans-disciplinary level of competency to improving patient safety (Valentin, Capuzzo, Guidet, Moreno, Dolanski, Bauer, & Metnitz, 2006). Basically to ensure patient safety especially is considering a progressive more important for intensive care unit practitioners (Peris, 2011), world health organization (WHO) has defined patient safety (PS) as “the absence of preventable harm to a patient during the process of health care” (WHO, 2012). In providing nursing care in critical care units for unstable patients in need to more and specific safety measures (American Nurse Association, 2011).

Patient safety is a key component of hospital performance and improving ICU staff nurses’ performance remains an ideal that every organization strives to achieve this goal, as well as, when providing the workers with new staff development strategies make their work of a high quality and potential errors are minimized. However training program conducted to nursing staff by using the available resources is consider the best way to improve patient safety (Garrouste-Orgeas et al., 2008; Despins, 2009). In Hewsons’ study, 2007 founded that when assessing an “unsafe ICU” there are six domains include teamwork climate, job satisfaction perception of management, safety climate, working conditions, and stress recognition (Hewson, 2007).

Critical care nurses should use a variety of methods to ensure patient safety, often relying upon their knowledge of the patient and personal relationships with other nurses and staff to identify and prevent errors (Walston, Al-Omar, Al-Mutari, & Int, 2010; Alamry, Al-Owais, Al-Dorzi, Noushad, & Taher, n.d.). On the other hand high nursing workload and fatigue have been identified as major negative contributors to patient safety (Orgeas, Timsit, & Vesin, 2010). So providing safe and error-free care is the priority of all health care professionals (Hartel & Barz, 2011).

Also safety care has been strongly associated with nurses and patient satisfaction, however with a safe culture, nursing staff are guided by an organization wide commitment to ensure safety, whereas each member upholds their own safety norms and those of their coworkers (Padilha, 2012). Therefore understanding that culture changes incrementally and that nursing staff must live a culture of safety, we sequentially implemented a safety program to engage and empower staff to identify and eliminate patient safety hazards. Additionally training nursing staff to use the complex ICUs equipments would lead to improving patient safety, because lack of this knowledge could lead to incorrect use of the equipment thus not delivering the expected effect (Helmreich & Merrrit, 1998).

Alamry et al. (2007) studied the Incident reporting at a tertiary care hospital in Saudi Arabia and suggested that additional prospective research is needed to further understand patient safety. There was no such this program applied in KSA. Furthermore Almutairis’ studied and added that the nurses’ perceptions of the clinical safety culture in Saudi Arabia environment was unsafe. So the staff development strategies through implementing patient safety educational program can minimize the medical errors and improve patient outcome in hospital.

### 1.1 Objectives of the Study

The objective of the present study was to:

-Implement staff development strategies through patient safety educational program.
-Evaluate the effectiveness of implementing patient safety strategies to ICUs staff nurses.

### 1.2 Research Hypothesis

-We hypothesized that better adherence to program guidelines would result in improving patient outcomes.
-Staff nurses’ knowledge, skills and attitudes about patient safety measures will be improved after implementing program

## 2. Subjects and Methods

### 2.1 Design and Settings

The study was carried out using a quasi experimental design. The settings included the intensive care units (ICUs) at General Mohail Hospital and National Mohail Hospital, King Khalid University, Saudi Arabia. The study was conducted from March to June 2012.

### 2.2 Sample

A convenience sample of all prevalent nurses at three shifts in the aforementioned settings during the study period was recruited. We arranged with nurse directors for suitable time which all nurses were available. The total number of the nurses was 50, their age ranged between 25–40 years, the educational level of RNs varies at ICUs, and these include RNs with two-year and three-year diploma, four-year baccalaureate.

### 2.3 Instruments

**Tool I**

The Safety Attitudes Questionnaire (SAQ)—ICU version, The SAQ was specifically to measure safety culture at both the individual and group level (e.g. at the ICU or job category level). The SAQ is consisted of 60 item survey instrument to assesses safety attitude across six factors—perceptions of management, job satisfaction, medical errors, stress recognition, teamwork climate and safety climate. Each item is measured on a 5-point Likert scale (Agree Strongly to Disagree strongly), which is then converted to a 0–100 scale. Each factor score equals the mean score of its component survey items. A positive score is defined as ≥75 out of 100. To calculate as a positive score for a given factor, the survey respondent must answer, on average, Agree Slightly or higher to all related items.

**Tool II**

Pre-post test exam was used to assess the ICU nurses knowledge regarding the procedures which may contribute in patient safety measures. The questionnaire was divided to five dimensions (8 dimensions for documentation, 6 dimensions for patient safety measures, 6 dimensions for fall and restraint, 4 dimensions for medical errors, and for infection control was 6 dimensions. The marks were ranged between 1- 4 (Poor- Excellent).

**Tool III**

Pre-post procedures check list to assess ICU nurses practice that can affect on patients safety. Each procedure had separate check list for suctioning, wound care, wearing sterile gown, ect. Each check list hade 10 score and was observed before and after implementation of the program.

### 2.4 Ethical Considerations and Human Rights

The study proposal was approved by Dean Ship of scientific Research at King Khalid University and administration from both hospitals. Nurses were informed about the purpose of the study and about their rights to refuse or withdraw at any time. The study findings would lead to beneficence in terms of improvement of patient safety measures.

### 2.5 Statistical Analysis

Data entry and statistical analysis were done using SPSS 18.0 statistical software package. Nurses’ Demographic data are reported as numbers (percentage) for qualitative data. Pearson correlation analysis was used for assessment of the relations between six main patient safety dimensions. The mean numbers were conducted to dimensions. Statistical significance was set at a p<0.05.

## 3. Results

The program was implemented on 50 staff nurses in different ICUs. Their age ranged between 25-40 years, with a mean of 1.7400±0.72309, the majority of the nurses were staff nurses 39 nurses, while 11 were head nurses and charge nurses, 42(62.7%) nurses were working in mixed ICU, and remaining 8(12%) were working in medical and neonate ICUs. The years of experiences in ICU ranged between one year 11(16.4) to 10 years 20(29.8) with mean of 2.4000±1.06904, most of them (68%) were working in variable shift, while 32% were day shift only (Table 1).

**Table 1:** Socio-demographic characteristics of ICUs staff nurses (n=50).

Demographic Data	Saudi ICU staff nurses (n=50)			

	N	%	Mean	Standard Deviation
**Job Position**				
CCU RN	39	78.0		
Nurse Manager	4	8.0	2.6400	0.7217
Charge Nurse	7	14.0		
**Type of ICU**				
Neonatal	39	78.0	1.2400	0.5911
Medical	4	8.0		
Mixed	7	14.0		
**Age group**				
36-40 Y	8	16.0		
30-35 Y	21	42.0	1.7400	0.72309
25-29Y	21	42.0		
**ICU job status**				
Contract	30	60.0	2.8600	1.42871
Part-Time	3	6.0		
Full Time	17	34.0		
**Total Years of Experiences**				
10.5-15 Y	13	26.0		
5.5-10 Y	7	14.0	2.5200	1.03490
1.5-5 Y	23	46.0		
one year or less	7	14.0		
**Experiences In ICU**				
10.5-15	11	22.0		
5.5-10 years	9	18.0		
1.5-5	19	38.0	2.4000	1.06904
one year or less	11	22.0		
**Usual Shift**				
Variable shift	34	68.0	3.0400	1.41364
Day	16	32.0		

Table 2 Shows that statistically significant relation was revealed between safety climate and job satisfaction among nurses in the study sample (p=0.001). However; there was negative correlation between the safety climate with stress recognition (p=0.889), and positively correlated to the medical errors. Conversely, the medical errors are highly significant correlated to perception of management (p=0.000). The strongest correlation is between teamwork climate and all patient safety dimensions.

**Table 2:** Correlation among main domains in patient safety

		Job satisfaction	Safety climate	Medical errors	Perception of management	Stress recognition	Teamwork climate
Job satisfaction	Pearson Correlation	1	.448**	.522**	.332*	.237	.742**
	Sig. (2-tailed)		.001	.000	.019	.098	.000
	N	50	50	50	50	50	50
Safety climate	Pearson Correlation	.448**	1	.473**	.457**	-.020-	.640**
	Sig. (2-tailed)	.001		.001	.001	.889	.000
	N	50	50	50	50	50	50
Medical errors	Pearson Correlation	.522**	.473**	1	.538**	.026	.699**
	Sig. (2-tailed)	.000	.001		.000	.859	.000
	N	50	50	50	50	50	50
Perception of management	Pearson Correlation	.332*	.457**	.538**	1	-.012-	.453**
	Sig. (2-tailed)	.019	.001	.000		.935	.001
	N	50	50	50	50	50	50
Stress recognition	Pearson Correlation	.237	-.020-	.026	-.012-	1	.383**
	Sig. (2-tailed)	.098	.889	.859	.935		.006
	N	50	50	50	50	50	50
Teamwork climate	Pearson Correlation	.742**	.640**	.699**	.453**	.383**	1
	Sig. (2-tailed)	.000	.000	.000	.001	.006	
	N	50	50	50	50	50	50

**. Correlation is significant at the 0.01 level (2-tailed).

*. Correlation is significant at the 0.05 level (2-tailed).

As observed in Table 3 the respondents mean ±standard deviation to work condition were (438.50±74.8), while the teamwork climate means ±standard deviation were 1217.7± 239.1.

**Table 3:** Comparison between respondents’ mean & standard deviation of six patient safety dimensions

Domains	Mean	Std. Deviation	Minimum	Maximum
Medical Errors	438.5000	74.78029	250.00	550.00
Teamwork climate	1217.0601	239.08009	675.00	1600.00
Perception of management	609.5600	144.38217	253.00	950.00
Safety climate	730.1200	106.81562	527.00	877.00
Stress recognition	509.5000	117.24843	225.00	675.00
Job satisfaction	392.5000	96.39550	200.00	575.00

Table 4 illustrates that comparison between the frequencies distribution of pre-post test grade with 34.0% of respondents had poor in pre test, in contrast the 60% got high marks (48% very good, 12% excellent), with mean 1.70±3.70 for pre test and for post test mean .544±.707. In addition, there was positive significant correlation p=0.296 between pre-post test.

**Table 4:** Comparison between frequencies, mean, standard deviation and correlation of pre- post test

Grad	Pre-Test		Post-Test	

	N	%	N	%
Poor	17	34.0	-	-
Pass	31	62.0	1	2.0
Good	2	4.0	19	38.0
Very Good	-	-	24	48.0
Excellent	-	-	6	12.0
Mean	1.7000		3.7000	
Std. Deviation	.54398		.70711	
P	.296			

Total	50		100	

As observed in Table 5 there was positive significant correlation p=0.060 between pre-domining and removing personal protective equipment and post checklist, also the correlation between performing hand washing pre and post was significant p=0.032. On the other side there was negative correlation p= 0.778- between wearing sterile gown before and after the program.

**Table 5:** Correlation between nursing procedures in ICU before and after implementing the program

Pre checklist	Post checklist									

	1	2	3	4	5	6	7	8	9	10
Domining and removing personal protective equipment	.060									
Performing hand washing		.032								
Domaining sterile gloves			.332							
Administration of subcoutenouse injection				.301						
Intramuscular injection					.900					
Wound care						.753				
Sterile gown							-.778-			
Opening sterile packs								.133		
Intravenous injection									.918	
Suctioning										.540

*. Correlation is significant at the 0.05 level (2-tailed).

1- domining and removing personal protective equipment post, 2- performing hand washing, 3- domaining sterile gloves, 4- administration of subcoutenouse injection 5- intramuscular injection, 6- wound care, 7-sterile gown, 8-opening sterile gown,9- intravenous injection, and10-Suctioning.

Table 6 explains; that no significant correlation (p=.653) between years of experience and respondents knowledge at pretest, while at post test there was significant correlation (p=0.142). Also there was no significant correlation between pre- post test and experience in ICU.

**Table 6:** Correlation between years of experience and pre-post test.

		P
	Pre-test	Post-test
Total years of experiences	.653	.142
Experience in ICU	.538	.576

*. Correlation is significant at the 0.05 level (2-tailed).

## 4. Discussion

Ensuring ICU patient safety regard as a key component of hospital performance, also it is a focus of increasing attention at all levels of the health care system (Walston et al., 2010).

Of great concern, was the educational program impact on the enhancing nurses’ performance in turn can improve ICU patient safety? In the present study it was revealed that the ICU nursing staff had low knowledge of patient safety through pre-test was done before our intervention, with a pass rate of 62% for the baseline test. Nurses’ knowledge improved after they had attended the program, as indicated by higher mean scores on the post test (3.7000±.70711). This result in the same line as those reported by Mccaffrey, 2012 that mentioned the formal educational program can assesses in developing nurses knowledge and practices in ICUs (Mccaffrey, Hayes, Cassell, Miller-reyes, Donaldson, & Ferrell, 2012). In addition that ICU nursing staff unit-based programs may have a significant (p=0.05) impact on patient safety climate (Shojania & Thomas, 2013).

Moreover, this finding could be attributed to indicate that no significant difference regarding knowledge level between staff nurses and years of experiences, that may be due to lack of training course in ICU, shortage of staff nurses, lack of self learning. These findings support the results of Said 2013 that mentioned that no association between knowledge and years of working experience (p- value 0.34) for ICUs staff nurses. However, nurses who had been in their current position for more than 5 years were less knowledgeable than nurses whose appointments were more recent (Said, 2013).

Contradictory with our result Jansson 2013 pointed that more experienced nurses performed significantly better than their less-experienced colleagues (*p* = 0.029) (Jansson & Kokko, 2013).

Regarding nurses job satisfaction that there was a highly significant relationship between job satisfaction and teamwork climate, that mean the effective relationship and teamwork can enhance nurses satisfaction enrolled improve patient safety. In the same line, collaborative interdisciplinary relationships were one of the most important predictors of job satisfaction for all healthcare providers (Chang, Ma, Chiu, Lin, & Lee, 2009; DiMeglio et al., 2005). In addition that within ICUs nursing staff; the higher level of teamwork leads to greater job satisfaction with current position and occupation (Kalisch, Lee, & Rochman, 2010).

Furthermore the result of this study revealed that the domain of teamwork had highly significant relationship with all domains; that represent when staff nurses working together they were clearly important contributor to satisfaction as are perceptions of staffing adequacy (Kalisch, 2010). Also when ICUs nurses communicate effectively; this can improve of safety culture in the unit, In addition teamwork has been associated with a higher level of quality of care (Gifford, Zammuto, & Goodman, 2002), an increase in patient safety (Grumbach & Bodenheimer, 2004), greater patient satisfaction with their care (Baker, Gustafson, Beaubien, Salas, & Barach, 2005), more productivity, and a decreased stress level (Rondeau & Wagar, 1998).

On the other hand; medical errors numerous studies have documented that despite high profile calls for enhancing safety culture in ICU (Rondeau & Wagar, 1998; Institute of Medicine, Health Care in America, 1999), also that medical errors have been reported to be a main cause of death in hospitalized patients (Hartel & Barz, 2011). Although high work load may reduce the attention devoted by a nurse to safety critical roles, thus leading for medical errors and unsafe patient care (Kutney-Lee et al., 2009), controversy with our findings; there was no significant correlation between the medical errors and workload; thus may be due to the small size of selected ICUs, but nurses responses to medical errors was negatively. Unfortunately medical errors can have negative effect for both patient and nurses.

Given these observation regarding safety culture, corrective effort might focus safety climate, the findings of our study represented that no one was expressed positively to safety culture, thus highlight the potential importance of a focus on how to make organization culture more safety. In the same line of our result there is significant correlation between safety culture and teamwork climate, so when the ICU staff nurses working at safety culture that can foster patient satisfaction and patient centered care and encourage them to deal with uncertainty (Siegele, 2009).

Although there is no significant relationship between stress recognition and most of domains like job satisfaction, safety climate, medical errors and perception of management, controversy of our result that the high workload in the form of stress may reduce the attention devoted by a nurse to safety-critical tasks, thus creating conditions for errors and unsafe patient care (Reason, 1990; Vincent, Taylor-Adams, & Stanhope, 1998).

Ultimately our study adds to the body of knowledge about ICU patient safety attitude by demonstrating the relative consistence of domain scores across a variety of ICUs.

## 5. Conclusion

Improvements were observed in safety climate, teamwork climate, and nurse turnover rates in ICUs after implementing a safety program. On the heels of this improvement; nurses’ total knowledge, skills and attitude were enhanced regarding patient safety dimensions. As our result, we improved staffs’ perception about patient safety and eliminate ICUs medication errors, and potentially nurse turnover. These findings suggest that healthcare organizations can develop patient safety and provide evidence for the business case for safety. In closing, this program provides simple tools to improve the culture of safety, a common metric to evaluate the culture of safety, a standard approach to improvement, and a system to publicize results within the organization. Continuous educational program for ICUs nursing staff through organized in-service training is needed to increase their knowledge and skills about the importance of improving patient safety measure. Emphasizing on effective collaborative system also will improve patient safety measures in ICUS.
